# MOBIlity assessment with modern TEChnology in older patients’ real-life by the General Practitioner: the MOBITEC-GP study protocol

**DOI:** 10.1186/s12889-019-8069-2

**Published:** 2019-12-19

**Authors:** Mareike Münch, Robert Weibel, Alexandros Sofios, Haosheng Huang, Denis Infanger, Erja Portegijs, Eleftheria Giannouli, Jonas Mundwiler, Lindsey Conrow, Taina Rantanen, Arno Schmidt-Trucksäss, Andreas Zeller, Timo Hinrichs

**Affiliations:** 1 Department of Sport, Exercise and Health, Division of Sports and Exercise Medicine, University of Basel, Birsstrasse 320 B, 4052 Basel, Switzerland; 2 Geographical Information Systems Unit, Department of Geography, University of Zürich, Zürich, Switzerland; 3 Gerontology Research Center and Faculty of Health Sciences, University of Jyväskylä, Jyväskylä, Finland; 4 Center for Primary Health Care, University of Basel, Basel, Switzerland

**Keywords:** Aging, General practice, Multimorbidity, Walking speed, Mobility limitation, Smartphone, Geographic information systems, Inertial sensors, Health promotion, Spatial behavior

## Abstract

**Background:**

Mobility limitations in older adults are associated with poor clinical outcomes including higher mortality and disability rates. A decline in mobility (including physical function and life-space) is detectable and should be discovered as early as possible, as it can still be stabilized or even reversed in early stages by targeted interventions. General practitioners (GPs) would be in the ideal position to monitor the mobility of their older patients. However, easy-to-use and valid instruments for GPs to conduct mobility assessment in the real-life practice setting are missing. Modern technologies such as the global positioning system (GPS) and inertial measurement units (IMUs) - nowadays embedded in every smartphone - could facilitate monitoring of different aspects of mobility in the GP's practice.

**Methods:**

This project’s aim is to provide GPs with a novel smartphone application that allows them to quantify their older patients’ mobility. The project consists of three parts: development of the GPS- and IMU-based application, evaluation of its validity and reliability (Study 1), and evaluation of its applicability and acceptance (Study 2).

In Study 1, participants (target *N* = 72, aged 65+, ≥2 chronic diseases) will perform a battery of walking tests (varying distances; varying levels of standardization). Besides videotaping and timing (gold standard), a high-end GPS device, a medium-accuracy GPS/IMU logger and three different smartphone models will be used to determine mobility parameters such as gait speed. Furthermore, participants will wear the medium-accuracy GPS/IMU logger and a smartphone for a week to determine their life-space mobility. Participants will be re-assessed after 1 week. In Study 2, participants (target *N* = 60, aged 65+, ≥2 chronic diseases) will be instructed on how to use the application by themselves. Participants will perform mobility assessments independently at their own homes. Aggregated test results will also be presented to GPs. Acceptance of the application will be assessed among patients and GPs. The application will then be finalized and publicly released.

**Discussion:**

If successful, the MOBITEC-GP application will offer health care providers the opportunity to follow their patients’ mobility over time and to recognize impending needs (e.g. for targeted exercise) within pre-clinical stages of decline.

## Background

### Multimorbidity and health care for older adults

Demographic transformation and increasing life expectancy in industrialized countries comes along with a growing number of older adults suffering from chronic health conditions, such as osteoarthritis, coronary heart disease, diabetes mellitus, or dementia [1]. The proportion of patients with “multimorbidity”, usually defined as the co-occurrence of at least two chronic conditions [2], grows accordingly. Figures on the prevalence of multimorbidity among over 65-year-olds vary between 40 and 85% [2–5]. Multimorbidity is strongly associated with poor clinical outcomes, including reduced quality of life [6], increased risk of inappropriate medication with major side-effects [5, 7] as well as high mortality and disability rates [8, 9]. The burden of disease is substantial for the affected patients and their relatives, and also for the healthcare system [10–12].

The simple definition of multimorbidity hardly accounts for the complex relationships between concurrent chronic diseases and the difficulties in managing multimorbid patients. It has been recognized that these patients’ needs are insufficiently served by traditional ways of health care provision [12–14]. One reason is the disease-oriented rather than integrated approach towards managing patients with multimorbidity [15, 16]. Disease-specific guidelines are often contradictory and impractical when applied to multimorbid patients. Disease-oriented management often results in polypharmacy and knowledge of potential side effects, hazards and harm of interventions is insufficient [15]. This lack of knowledge is even more striking, as it is not clear whether traditional improvements in outcomes, for example mortality, are attainable and desirable in patients with multimorbidity [16]. Patient and relative preferences are frequently unmet, as are patient-oriented outcomes such as improving physical functioning and maintaining independence [17, 18]. There is even evidence that the association between multimorbidity and mortality is lost when adjusted for functional impairment [19]. Hence, there is increasing awareness of the importance of physical functioning as a basic integrator of older adults’ health and as a major health outcome; optimizing functional status has been recognized as a central goal for all persons with chronic illness [20].

### Mobility

“Mobility” is a central element of physical functioning [21, 22]. It has been defined comprehensively as “the ability to move oneself (either independently or by using assistive devices or transportation) within environments that expand from one’s home to the neighbourhood and to regions beyond” ([23], p. 444). Hence, measures that have been used to characterize a person’s mobility include tests of “physical function” [24], and assessments of “life space” [25].

Lower extremity physical function can be measured by simple tests such as stop-watch measured gait speed, balance measures such as maintaining the tandem stand for 10 s, or simple assessments of muscle power such as timing of 5 sit-to-stand cycles [24, 26]. In relation to a person’s mobility, gait speed is one of the most central functional parameters. In prospective studies, poor physical function as well as reduced gait speed alone have been shown to be highly predictive of falls [27, 28], dependency in basic activities of daily living [29–31], healthcare utilization [32–34], and mortality [35, 36]. Reduced physical function has been shown to foster social isolation [37], anxiety and depression [38], and to be associated with an overall reduced quality of life [39]. Based on the existing evidence from large-scale prospective studies, clinical cut-offs for functional measures, indicating an increased risk for falls or an increased risk for mobility disability, have been established [40–42], so that these measures have found their way into routine geriatric inpatient care and rehabilitation settings.

Life-space, the “spatial extent in which a person moves within a specified period” ([43], p. 155), encompasses “the interaction between intrinsic capabilities of the person and the demands of the extrinsic environment” ([43], p. 155). It has been shown that restricted life-space is predictive of disability in activities of daily life (ADL) [44], nursing home admission [45] and mortality [46]. Until now, epidemiological studies mostly relied on questionnaires to measure life-space. However, the geospatial information gained from questionnaires is rather crude. As an example, the University of Alabama at Birmingham (UAB) Study of Aging Life-Space Assessment (LSA) assesses the extent of a person’s movement (within the past 4 weeks) categorized into 5 spatial levels, ranging from the participant’s bedroom to places outside the participants’ home town [25]. While the independent predictive value of life-space on different health-related outcomes has been shown repeatedly [45–48], recommendations on clinical cut-offs are currently sparse (e.g .[ 49]) and measures of life-space have not yet found their way into clinical practice.

### The necessity to assess mobility

Measures of functional status have been suggested to inform health care payment systems [50] and they are central components of decision trees advocated by current fall prevention guidelines [27, 41]. In early stages of decline, physical function can be stabilized or even reversed by targeted intervention such as exercise programmes [51]. Life-space, and thereby participation in social life, can be maintained by adapting the environment to the patients’ needs (e.g. by providing assistive devices, adapting patients’ homes or providing social support). Similar to chronic illness, physical functioning is dynamic in nature [52]; mobility should therefore be routinely monitored to account for the dynamic interactions between physiological systems and the environments of daily life over time. It has even been suggested that functional status should be considered the “sixth vital sign” in addition to the conventional vital signs (body temperature, pulse etc.) [20, 53, 54].

### The general practitioner’s practice – an opportunity for targeting older adults’ mobility

As mentioned, physical function tests have found their way into routine geriatric inpatient care and rehabilitation settings, but general practitioners (GPs) do not routinely assess physical function as part of their older patients’ management [55, 56]. This deficiency in administering assessments might occur for a number of reasons: physical function assessments (e.g. the measurement of 4 m gait speed) require standardized equipment, space and training; they are time- and therefore cost-intensive. There is a lack of reimbursement and particularly in patients with multiple chronic conditions, GPs have to weigh the realization of functional assessments against other preventive and therapeutic services [57, 58]. These considerations are in contrast to the ideal position that GPs would have to monitor their older patients’ mobility. GPs are among the few persons who have regular access to community-dwelling and mobility-limited older adults [59, 60]; they often have established long-lasting, ongoing and trusting relationships with their older patients [61]. GPs are also able to appraise the results of a mobility assessment against the background of their patients’ overall health status and medical history.

### New opportunities offered by modern technologies – observing mobility in the “real-life” setting

Modern technologies such as Global Navigation Satellite Systems (GNSS) – including various satellite systems such as GPS, GLONASS, Galileo and Beidou – and inertial measurement units (IMUs) – including accelerometer, gyroscope and magnetometer – offer the chance to implement measures of mobility (physical function and life-space) into routine primary health care and to follow-up on older patients’ mobility over time. In contrast to traditional assessment tools, these technologies allow for observation of older adults function in real-life settings and in interaction with their environment [62, 63]. It has been criticized in the past that laboratory-based functional assessments only have limited value in predicting older adults’ real-life mobility behaviour [64]. GNSS technology and IMUs are nowadays embedded in every modern smartphone, a device that has become popular and shows much promise in improving the health care of older adults. High availability, objectivity and low additional costs might facilitate widespread future use.

While high-end, handheld GPS devices can achieve decimetre accuracy, typical consumer GPS devices or smartphones will achieve best-case accuracies of 2–3 m under normal conditions [65]. Latest smartphone models are also meant to achieve decimeter-level positioning accuracy in post-processing mode, due to the ability to use dual frequency GPS/GALILEO [66]. That is, consumer-level products are starting to reach a positioning accuracy level where accurate and robust movement parameter derivation (e.g. distance, speed, acceleration etc.) is becoming feasible, particularly for distances that exceed the GPS error by at least one order of magnitude, that is, several dozens to some hundred meters. So far, however, the use of GPS-derived movement parameters to quantify physical performance has mainly been limited to team sports [67, 68]. In the health sciences, most applications have been confined to using GPS fixes to estimate individuals’ life-spaces [69], often linking these to active transport and body weight [70]. There are few reports on applications of GPS-derived movement parameters in patient populations. A limited number of studies have shown the reliability and applicability of GPS-derived measures in a sample of patients with peripheral arterial disease [71, 72].

Locomotion speed can already be accurately extracted from IMU readings over shorter distances [73, 74]. Since GPS and IMUs are standard components of contemporary smartphones, there is potential for smartphone-based measurements to replace or complement traditional – usually stopwatch timed – walking tests over short distances (e.g. 10 m). In contrast to GPS devices, IMUs do not require satellites visibility, i.e. they can be used both indoors and outdoors. On the other hand, GPS is the optimal method to determine locations over longer periods of time, and therefore the optimal method to assess life-space mobility.

### Steps to be taken

Though the sources quoted above have demonstrated the potential of GPS- and IMU-derived measures, they still have to be adapted to the new context and tested for their reliability, validity, applicability and acceptance before they can be routinely used by GPs to monitor their patients’ mobility (physical function and life-space). There is also a need to evaluate how GPS- and IMU-derived mobility measures relate to traditional physical function tests.

## Methods and design

### Aims and objectives

The overall goal of the MOBITEC-GP project is to provide GPs with the possibility to assess their older patients’ mobility using a collection of different mobility parameters (related to physical function and life space).

The aims of the project are:

1. To develop an easy-to-use GPS- and IMU-based smartphone application that allows GPs to quantify and appraise their older patients’ mobility (“Development”);
2. To evaluate the application’s validity and reliability (“Study 1”);
3. To evaluate the new tools’ applicability and acceptance among GPs and patients (“Study 2″).

**Fig. 1:**
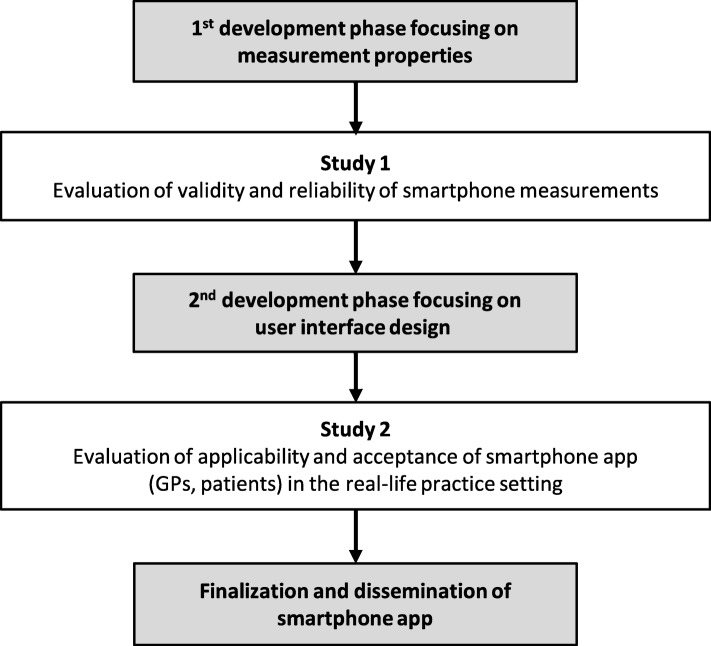
Iterative process of development and evaluation of the new smartphone application

An iterative development and evaluation process is required to fulfil all aims; it is illustrated in Fig. 1. The development phases include requirement analyses among GPs, patients and experts. While the first development phase focuses on the app’s measurement properties, the second development phase focusses on user interface design. The app will be specifically designed to create a patient-GP partnership, i.e. the patient collects data of daily functioning and the GP interprets and explains the data to the patient, and plans the necessary measures (such as referral to a comprehensive fall risk assessment or to an exercise programme, home adaptations or provision with adaptive devices) together with the patient.

Specific objectives of Study 1 are:

1. To assess the test-retest reliability of GPS/IMU-derived physical function measures (smartphone, medium-accuracy GPS/IMU device, high-end GPS device; see section ‘Measurements in Study 1’ below) obtained during standardized short-distance mobility tasks (10 m and 50 m walks; “short distance controlled condition”) and their validity against “gold standard” (timing, video-recording).
2. To assess the test-retest reliability of GPS/IMU-derived physical function measures (smartphone, medium-accuracy GPS/IMU device, high-end GPS device) obtained during a standardized long-distance mobility task (400 m walk on a given track; “long-distance controlled condition”) and their validity against “gold standard” (timing, video-recording).
3. To assess the test-retest reliability of GPS/IMU-derived physical function measures (smartphone, medium-accuracy GPS/IMU device, high-end GPS device) obtained during a semi-standardized mobility task (30-min stroll in the park; “semi-controlled condition”) and their concurrent validity (against traditional physical function tests).
4. To assess the test-retest reliability of GPS/IMU-derived life-space measures (smartphone, medium-accuracy GPS/IMU-device) during a one-week measurement period around participants’ homes (uncontrolled “real-life” condition) and their concurrent validity (against traditional physical function tests).
5. To assess whether measurement properties (validity, reliability) vary by key participant characteristics (including demographic characteristics, anthropometrics, and physical function measures).
6. To assess whether measurement results (physical function and life-space) vary by key participant characteristics (including demographic characteristics, physical activity level, gait efficacy, fall status, and morbidity) to generate hypotheses for subsequent large-scale epidemiological studies.
7. To assess the inter-instrument agreement between a high-end GPS device, a medium-accuracy GPS/IMU device and three different smartphone models, including agreement between the different smartphone models and agreement between smartphone wearing locations.
8. To define cut-offs for indicators of different mobility levels against established clinical cut-offs based on traditional physical function measures (from existing epidemiological studies and current fall-prevention guidelines).

Specific objectives of Study 2 are:

9) To assess the clinical applicability of unsupervised GPS/IMU measurements with the smartphone (fitted with the new application) in the real-life general practice setting.
10) To assess usability and acceptance by GPs and patients.
11) To assess participant characteristics that are associated with the proportion of clinically usable unsupervised GPS/IMU measurements.

### Design

Study 1 and Study 2 will both be observational. Study 1 will be a validity and reliability study. At baseline (T_0_), a battery of tests (visit at study center) as well as a 1-week measurement around participants’ homes will be performed. Cross-sectional analyses of baseline values will be used to evaluate validity of the GPS and IMU-based measurements as well as the inter-instrument agreement between devices of different accuracy levels as well as between different smartphone models. Participants will be assessed again 1 week after the initial assessment (T_1_) to evaluate test-retest-reliability of the GPS/IMU measurements. T_1_ includes a visit at the study center and a 1-week measurement around participants’ homes.

Study 2 will be an applicability study: Patients equipped with a smartphone (fitted with the new application) will independently perform mobility measurements around their homes for 1 week. The proportion of technically satisfactory GPS/IMU recordings will be determined. Reasons for unsuccessful recordings will be assessed and analysed. Usability and acceptance by GPs and patients will be evaluated.

### Inclusion criteria

Both studies target community-dwelling older patients from general practice with multiple chronic conditions, aged 65 or above. The target number of participants for Study 1 is 72, the target number of participants for Study 2 is 60. Participants have to be diagnosed (self-report) with at least two of the following chronic diseases (according to the “Self-Administered Comorbidity Questionnaire” (SCQ) [75, 76]): heart disease, high blood pressure, lung disease, diabetes, ulcer or stomach disease, kidney disease, anemia or other blood disease, cancer, depression, osteoarthritis, degenerative arthritis, back pain and/or rheumatoid arthritis. Participants have to be able to perform a 30-min outdoor walk at their own pace, with or without breaks, with or without walking aid, but without the help of another person (self-report). Persons who are incapable of judgment and persons who are unable to follow procedures or have insufficient knowledge of the German language will be excluded. Participants will have to provide written informed consent.

### Recruitment

The recruitment strategy will be twofold. On one hand, participants will be recruited through GP practices. Patients who attend their GP within a given timeframe, are capable of judgement and aged 65 or above will be asked by the GP for their general interest in participating in a study on “mobility”. If they agree they will be informed about the study and assessed for eligibility by a research team member. All eligible patients will be invited to participate. On the other hand, participants will be recruited through presentations about the project in local senior citizen gatherings, individual invitations to persons who expressed interest in participating in studies from our institute, as well as handing out information brochures about the study and individually approaching older adults in settings such as pharmacies, churches, and senior sports groups.

GP practices will be recruited from a network of practices associated with the Center of Primary Health Care, University of Basel.

### Ethical considerations and ethical approval

The performed measurements will only include everyday tasks (such as walking at preferred, habitual pace or rising from a chair), so that they do not involve an increased cardiovascular or musculoskeletal risk compared to everyday activity. During the walking tests, participants will be allowed to take a break at any time.

The new smartphone application will be designed in a way that GPs are only provided with summary measures of physical function and life-space mobility. GPs will not have an insight into the raw movement data or locations that older adults have visited.

The research is carried out in compliance with the Helsinki Declaration. Data generation, transmission, storage and analysis of health related personal data will follow the current Swiss legal requirements for data protection. The project was approved by the Ethics Committee of Northwestern and Central Switzerland (EKNZ) (Reg.-No. 2018–02257).

### Measurements in study 1

#### GPS and IMU-based measures

GPS and IMU-based measurements will take place at two points in time (T_0_ and T_1_) at the study center. Four different mobility tasks including GPS and IMU-based measurements will be performed. Tasks a) to c) will take place at the study center; Task d) will take place in the week immediately after each visit around participants’ homes. The devices used in Study 1 will include a high-end GPS device (Trimble GeoExplorer 5 T, Trimble Inc., Sunnyvale, CA, USA); a medium accuracy GPS/IMU device (uTrail, CDD Ltd., Athens, Greece) and three different models of contemporary dual-antenna smartphones (Samsung Galaxy S8, Xiaomi Mi 8, Apple iPhone SE).

*Task a):* two supervised walks of 10 m and 50 m length, at self-selected, habitual pace on an outdoors athletics track (short-distance controlled condition). These tasks will be videotaped (Garmin VIRB XE, Garmin Ltd., Olathe, KS, USA) and timed (light barrier system; BROWER Timing Systems, Draper, UT, USA). Participants will be equipped with the high-end GPS device, the medium accuracy GPS/IMU device and the three smartphones. The three different smartphone models will be worn at three different locations (waist belt, sling bag, and neck pouch). This will be used to assess the influence of wearing location and model on the results.

*Task b):* a supervised 400 m walk at self-selected, habitual pace on an outdoors athletics track (long-distance controlled condition). This task will also be videotaped and timed. Again, participants will be equipped with the high-end GPS device, the medium-accuracy GPS/IMU device and three smartphones.

*Task c):* an unsupervised 30-min stroll in a park at self-selected, habitual pace without a given track (semi-controlled condition). Participants will be equipped with the high-end GPS device, the medium-accuracy GPS/IMU device and three smartphones.

*Task d):* a one-week measurement period around participants’ homes without any specified tasks (uncontrolled “real-life” condition). Participants will be equipped with an uTrail device, a smartphone (random choice of 1 of the 3 models) and a wrist-worn activity tracker (see section ‘Traditional physical function tests and physical activity’ below).

During all tasks, participants will be allowed to stop and rest any time. There will be no recommendation about minimum or maximum duration of stops. Weather conditions (temperature and cloud cover) will be documented at T_0_ and T_1_; these parameters will be considered for sensitivity analyses as they might affect reliability.

Data processing will include:

*Tasks a) and b):* Gait speed (average, average per section, and maximum), number of steps, number and duration of stops, and greatest distance between stops will be derived from the raw GPS/IMU data. Average gait speed (distance/time) and number of steps will also be derived from timing and video recording.

*Task c):* Gait speed (average, average per section, and maximum), number of steps, number and duration of stops, and greatest distance between stops will be derived from the raw GPS/IMU data.

*Task d):* Previously suggested approaches to derive summary measures of life-space from GPS data will be used (including convex hull and standard deviation ellipse) [77].

#### Traditional physical function tests and physical activity

In addition to the GPS/IMU-based measurements, physical function will also be assessed by a battery of traditional functional geriatric tests at T_0_ (before the GPS/IMU measurements) at the study center: short physical performance battery [24], single-leg stance [78], timed “Up & Go” [26], and grip strength (Jamar plus dynamometer, Sammons Preston, Bolingbrook, IL) [79]. Additionally, habitual physical activity will be assessed by the use of a wrist-worn activity tracker (vivofit 2, Garmin Ltd., Olathe, KS, USA) the week after T_0_.

#### Further measures

The following participant characteristics will be assessed within the baseline (T_0_) assessment (self-report): sex, age, residential area (urban, suburban or rural), living condition (alone or with someone else), socio-economic status (financial hardship and years of education), current walking ability (no walking aid, cane or rollator), frequency of falls (12-month recall) [80], sports participation [81], gait efficacy (modified Gait Efficacy Scale) [82, 83], perceived health status and disability (WHODAS 2.0 12-item version) [84], and chronic diseases (according to the inclusion criteria, see above). Height and weight will be measured by a trained assessor.

Participants will be given a diary for the one-week measurement, where they will note how often they left their homes, if they carried the GPS device and smartphone and if they have worn the activity tracker for at least 10 h a day.

### Measurements in study 2

#### Smartphone measurements

Study 2 will use smartphones fitted with the newly developed application. Patients will be instructed by a research team member on how to use the application. The application will be mostly self-explaining. Participants will be asked to take along the smartphone fitted with the application for a 1-week period. They should independently perform one continuous GPS/IMU measurement during a 30-min stroll in a park of their choice at self-selected, usual pace (to determine gait speed). Furthermore, participants’ position will be recorded by GPS over this period to determine life-space.

#### Further measures

Sex, age, height and weight, residential area (urban, suburban or rural; self-reported) and current walking ability (self-reported) will be documented. After finishing the 1 week measurements, participants will be contacted by a research member and they will be asked to rate the usability of the tool.

Furthermore, aggregated results will be presented to participating GPs. They will be asked to rate the usability of the tool and the usefulness of the obtained information.

### Statistical analyses

Participant characteristics (demographics, history of falls, chronic conditions etc.) will be analysed descriptively.

#### Study 1

##### Objectives 1 and 2

Test-retest reliability and validity will be assessed by calculating Intraclass Correlation Coefficients (ICCs) [85]. Agreement will also be assessed by performing Bland-Altman-Analyses [86]. In addition to ICCs, recently developed information-based measures to quantify reliability will be considered [87].

##### Objectives 3 and 4

Test-retest reliability will be assessed by calculating ICCs [85] (as well as information-based measures [87]). Again, Bland-Altman-Analyses will be performed [86]. Concurrent validity will be assessed by estimating correlations or associations (depending on the level of measurement of the respective parameters).

##### Objective 5

The above mentioned estimation procedures will be performed stratified by subgroups (based on demographic characteristics, anthropometrics, and physical function measures).

##### Objective 6

Measures of physical function and life-space will be analysed (descriptive statistics and 95% confidence intervals) for the total sample and stratified by subgroups (based on demographic characteristics, physical activity level, gait efficacy, and morbidity).

##### Objective 7

Inter-instrument agreement (as well as agreement between wearing locations) will be assessed by calculating ICCs [85] (as well as information-based measures [87]) and by performing Bland-Altman-Analyses [86].

##### Objective 8

Inter-rater agreement between levels of mobility based on traditional measures and the new smartphone-based measures will be assessed using Cohen’s Kappa [88]. Optimal cut-offs for the new measures will be chosen such that Cohen’s Kappa is maximized.

#### Study 2

##### Objective 9

The proportion (with 95% confidence interval) of satisfactory recordings (according to predefined criteria) [71] for the estimation of both life-space (continuous 1-week measurement) and physical function (30-min stroll in the park) will be calculated and presented for the total sample as well as stratified by relevant subgroups (defined based on findings of Study 1). Reasons for unsatisfactory recordings will be analysed. Furthermore, the obtained physical function and life-space data will be analysed descriptively for the total sample and stratified by relevant subgroups.

##### Objective 10

Acceptance by GPs and patients will be analysed by descriptive statistics.

##### Objective 11

Logistic regression models will be used to assess participant characteristics that are associated with the proportion of satisfactory recordings.

### Sample size calculation

#### Study 1

The primary analysis is the estimation of the ICC (95% confidence interval) between the T_0_ and T_1_ value of the GPS/IMU-derived average walking speed during the 30-min stroll in the park. To estimate an ICC of 0.9 with an expected 95% confidence interval width of 0.1, 61 patients will be necessary (underlying assumption based on results of Gernigon et al. [72]) [89]. Accounting for an anticipated drop-out rate of 15%, the target sample size is 72.

#### Study 2

The primary analysis is the estimation of the proportion of satisfactory GPS recordings from the 30-min stroll in the park. Assuming that 80% of GPS measurements will be successful (based on results of Gernigon et al. [71] (85%), but being more conservative due to the higher age and morbidity of participants in our study), 60 patients are needed to achieve an expected width for a 95% confidence interval of 0.2. We will use the Wilson interval which is an improvement over the traditional Wald-type interval [90]. No drop-outs are assumed for this study because there is no follow-up.

## Discussion

MOBITEC-GP will provide the prerequisites for a novel tool for primary health care, offering GPs the opportunity to routinely assess their older adult patients’ mobility and to recognize impending needs within pre-clinical stages of decline.

Making use of technologies that are embedded in smartphones offers a cost-effective opportunity as these devices are already available and no “extra device” is needed. The proportion of smartphone owners in the population is growing rapidly: between 2013 and 2018 it has increased from 70 to 90% in the general Swiss population [91]. Even though an US survey (2015) has shown that the proportion of smartphone users among those aged 65+ (27%) is still markedly lower than in the younger age categories (54% in those aged 50 to 64) [92], it can be expected that smartphone use in old age will increase in the future – substantially but not exclusively due to the aging of middle aged smartphone users [92]. A recent survey among patients after surgery showed that even though older patients were less likely to have a smartphone, they were just as interested and willing as their younger counterparts to engage with mobile health technologies [93].

Furthermore, the measurement as designed in MOBITEC-GP do not need supervision and thus are not personnel consuming. This will facilitate the use of these measures in primary health care as well as in future large-scale cohort studies, aiming to determine or confirm clinical cut-offs for targeted intervention. The advent of electronic medical records will provide an opportunity to link in situ mobility monitoring with medical records and to integrate algorithms that automatically detect critical values for fall risk and social isolation. The new tool could also provide an objective measure for other health care professionals, such as social workers, occupational therapists or physiotherapists, when life-changing decisions such as moving into a nursing home have to be made; or alternatively, ambulatory services have to be planned in order to enable older adults to stay in their own homes. Finally, the new tool might facilitate further research on the relationship between older adults’ functioning and their (geographical and social) environment.

## Data Availability

After completion of the project, data will be available from the corresponding author on reasonable request. The source code of the app as well as the analysis software will be publicly available as ‘open source’.

## Abbreviations

GNSS: Global Navigation Satellite Systems
GP: General Practitioner
GPS: Global Positioning System
ICC: Intraclass Correlation Coefficient
IMU: Inertial Measurement Unit
